# Antimicrobial Activity of *Lippia* Species from the Brazilian Semiarid Region Traditionally Used as Antiseptic and Anti-Infective Agents

**DOI:** 10.1155/2013/614501

**Published:** 2013-09-10

**Authors:** Cristiana da Purificação Pinto, Velize Dias Rodrigues, Fernanda da Purificação Pinto, Renata da Purificação Pinto, Ana Paula Trovatti Uetanabaro, Carla Santos Ribeiro Pinheiro, Suzana Ferreira Magalhães Gadea, Tânia Regina dos Santos Silva, Angélica Maria Lucchese

**Affiliations:** ^1^Laboratório de Química de Produtos Naturais e Bioativos, Departamento de Ciências Exatas, Universidade Estadual de Feira de Santana, Avenida Transnordestina S/N, Bairro Novo Horizonte, Campus Universitário, 44036-900 Feira de Santana, BA, Brazil; ^2^Laboratório de Microbiologia da Agroindústria, Universidade Estadual de Santa Cruz, Campus Soane Nazaré de Andrade, Km 16 Rodovia Ilhéus-Itabuna, 45662-900 Ilhéus, BA, Brazil; ^3^Departamento de Ciências Biológicas, Universidade Estadual de Feira de Santana, Avenida Transnordestina S/N, Bairro Novo Horizonte, Campus Universitário, 44036-900 Feira de Santana, BA, Brazil

## Abstract

*Lippia origanoides* Kunth, *Lippia alnifolia* Schauer, and *Lippia thymoides* Martius and Schauer are shrubs used in the traditional Brazilian medicine as antiseptics, as well as in the treatment of infectious diseases. This study was designed to investigate the antibacterial and antifungal activities of the methanolic extracts of these species, as new potential sources of antimicrobial drugs. The antimicrobial activity of methanolic extracts was investigated against resistant yeasts and bacteria by agar disk diffusion. Then, the MIC determination of the most active species and its fractions in hexane, dichloromethane, ethyl acetate, and water was performed. By the agar diffusion assay, all species were active against at least two microorganisms, giving evidence to support their use in the popular medicine. *L. origanoides* leaves exhibited the widest antimicrobial action, inhibiting the growth of two Gram-positive bacteria and two yeasts; this activity was also confirmed by the MIC evaluation. The fractionation of *L. origanoides* crude extracts improved the activity in spectrum and intensity. The results obtained in this study indicate that *L. origanoides* may be a promising alternative in the treatment of bacterial and fungal infections and in the seeking of new antimicrobial drugs.

## 1. Introduction

The development and spread of antimicrobial resistance is a worldwide concern, due to the negative impacts on public health [1]. Therefore, there is a growing need for novel drugs against bacterial, fungal, and viral infections. The traditional knowledge on the therapeutic potential of plants has been attracting scientific interest seeking new ways to control and treat many diseases caused by microorganisms [2–4]. 

The genus *Lippia* has a great number of medicinal species, such as *L. origanoides* Kunth, *L*. *alnifolia *Schauer, and *L. thymoides *Martius and Schauer, which are frequently used in folk medicine for the treatment of microbial diseases [5]. In Brazil, *L. origanoides* is popularly known as “salva-de-Marajó and “alecrim d'Angola.” The leaf infusions are used as a general antiseptic for the mouth, throat, and wounds, for the treatment of baby colic, diarrhea, indigestion, flatus, heartburn, nausea, vaginal discharges, menstrual complaints, and fever [5, 6]. *L. alnifolia *is also known as “alecrim-do-mato,” “pedrécio,” and “alecrim-de-vaqueiro.” The leaves are employed as a topic antiseptic against dermatitis and dandruff, as well as an oral antiseptic and in vaginal infections [7, 8]. Aerial parts of *L. thymoides*, known as “alecrim-do-mato,” are used in the treatment of skin infections [7].

Although the traditional use of these plants, studies supporting the antibiotic activity of *L. alnifolia* and *L. thymoides* are not found in the literature. Antimicrobial activity has been reported to *L. origanoides*, though it was related to the leaf essential oils [7, 9–11]. Therefore, a bioprospecting study was designed to investigate the antimicrobial activities of *L. alnifolia*, *L. origanoides,* and *L. thymoides* extracts from the Brazilian semiarid region against bacteria and yeasts, in order to select the most active species. 

## 2. Materials and Methods

### 2.1. Plant Material

Aerial parts of *L. alnifolia* (LA), *L. origanoides* (LO), and *L. thymoides* (LT) were collected in October 2006, in the city of Rio de Contas, BA, Brazil. Samples were identified by Dr. Tânia Regina dos Santos Silva, from Universidade Estadual de Feira de Santana. Voucher specimens were deposited at the UEFS herbarium, under the registry numbers HUEFS 112586, HUEFS 112591, and HUEFS 112597, respectively.

### 2.2. Extracts Preparation

The air dried and powdered stems, leaves, and flowers were macerated in methanol for 10 days at room temperature. Extraction was repeated at least five times, and the filtrates of all portions were combined. The solvent was removed by evaporation under vacuum on reduced pressure at 40–45°C using a rotary evaporator. Water residues were removed by lyophilization and packed in a glass pot to store in a refrigerator.

### 2.3. Fractions Preparation

The dry methanolic crude extracts (20 g) were resuspended in a methanol : water solution (1 : 1) and extracted with hexane (3 × 500 mL). After methanol removal by evaporation, the residual aqueous solution was subjected to a sequential partition with dichloromethane (3 × 500 mL) and ethyl acetate (3 × 500 mL). The solvent of each fraction, including the final aqueous fraction, was removed as described above.

### 2.4. Antimicrobial Activity

#### 2.4.1. Microorganisms

The antimicrobial effect of various extract samples was assayed against *Staphylococcus aureus* CCMB262 (streptomycin and dihydrostreptomycin resistant), *Staphylococcus aureus* CCMB263 (novobiocin resistant), *Bacillus cereus *CCMB 282, *Escherichia coli* CCMB261 (sulfonamide resistant), *Pseudomonas aeruginosa* CCMB268, *Candida albicans* CCMB266 (fluconazole and amphotericin B resistant), and *Candida parapsilosis* CCMB 288 (fluconazole and amphotericin B resistant) from the Culture Collection of Microorganisms of Bahia (CCMB). 

#### 2.4.2. Agar Disk Diffusion Method (ADD)

A screening for antimicrobial activity was performed by Agar Disk Diffusion method, according to the Clinical and Laboratory Standards Institute [12], with some modifications. Methanolic solutions of the extracts at 200 mg·mL^−1^ were sterilized by filtration through a 0.22 *μ*m membrane filter. Filter paper disks (*ø* 6 mm) were impregnated with 5 *μ*L of each extract solution (1 mg of crude extract/disk), and the methanol was evaporated at room temperature for 2 h. The microorganisms were grown on Agar Type 1 Himedia-RM 666 and Mueller Hinton Himedia-M391 broth. After 18 h (bacteria) and 36 h (yeast), the inoculum was adjusted to 5 × 10^5^ UFC·mL^−1^ and 1.5 × 10^8^ UFC·mL^−1^, respectively, in saline solution at 0.45%. Microorganism suspensions (100 *μ*L) were uniformly spread in Petri plates containing Mueller-Hinton Agar. The paper disks impregnated with the samples were placed on the surface of the agar. The plates were incubated at 28°C/48 h and 37°C/18 24 h for yeasts and bacteria, respectively. Inhibition zones (mm) were measured, and a positive result was considered with halos larger than 6.5 mm (disk diameter included). Nystatin (10 *μ*g/disk), erythromycin, and chloramphenicol (30 *μ*g/disk) were used as positive controls against yeasts and bacteria. The tests were done in triplicate.

#### 2.4.3. Minimum Inhibitory Concentration (MIC) Tests

The determination of the Minimum Inhibitory Concentration was performed as described in the CLSI [13, 14], with modifications. The extracts or fractions (42 mg) solubilized in DMSO 50% were sterilized by filtration through a 0.22 *μ*m membrane filter. In 96-well plates, 95 *μ*L of extracts solution and 95 *μ*L of Mueller-Hinton broth (2X concentrated) were conditioned in the first well, and the seriated dilutions were carried out in all subsequent wells. The range of evaluated extract concentration was from 21.05 mg·mL^−1^ to 0.01 mg·mL^−1^. Cultures of 18 h (bacteria) and 36 h (yeast) were collected to saline solution 0.45%, and 5 *μ*L of microorganism suspension at 9 × 10^6^ UFC·mL^−1^ and 5 × 10^5^ UFC·mL^−1^ (bacteria and yeasts, resp.) was added in each well. The microplates were incubated at 28°C/48 h for yeasts and 37°C/24 h for bacteria. An aqueous solution of 2,3,5-triphenyltetrazolium chloride (50 *μ*L at 5 mg·mL^−1^) for yeasts or rezasurin (30 *μ*L at 0,1 mg·mL^−1^) for bacteria was added in all wells. The result was read after 3 h of incubation, and red wells were considered an indication of microbial growth. The MIC was defined as the lowest concentration in which there was no visible growth after incubation. Controls with nystatin for yeasts (10 mg·mL^−1^) and chloramphenicol (20 mg·mL^−1^) for bacteria were made. Controls of the microbial strains viability, sample sterility, and water were also performed. The MIC of the DMSO solution in water (50%) was also determined. All tests were done in triplicate. Plant extracts with MICs ≤2.631 mg·mL^−1^ were considered active antimicrobial agents.

### 2.5. Phytochemical Screening

A phytochemical screening was performed on all fractions of *L. origanoides* by thin layer chromatography, according to the procedure described in the TLC Atlas “Plant Drug Analysis” [15]. 

## 3. Results and Discussion

The disk diffusion assay results are summarized in Table 1. All extracts have shown antimicrobial activity, inhibiting at least one of the evaluated microorganisms, as for the flowers and stems extracts of *L. thymoides*. A wide spectrum antimicrobial activity was noted with *L. origanoides* leaves, inhibiting the growth of two *S. aureus* strains, in addition to *C. albicans* and *C. parapsilosis*.

*S. aureus *CCMB262 was the most sensitive strain concerning theantibacterial effect of the extracts (seven out of eight extracts were active), whereas *E. coli* and *P. aeruginosa *were the least sensitive microorganisms (all extracts inactive). In general, the Gram-positive bacteria were the most sensitive to the tested methanolic extracts, with inhibition zones of 6.9 to 12.3 mm. This higher resistance of Gram-negative bacteria was expected, due to the polysaccharide outer membrane with a lower permeability and efflux pumps. This system prevents the accumulation of the antimicrobial agent inside the cell, thus impeding the substance from reaching its target or the concentration becoming lethal to the cell [16]. 

Despite the low permeability of the fungal cell walls, composed of 1,3-*β*-glucan and chitin polymers [17], an anticandidal activity was observed in *L. alnifolia* flowers, as well as in *L. origanoides* leaves. *C. parapsilosis *was the most sensitive yeast, which was inhibited by two out of eight extracts. 

*L*. *origanoides* was selected for further assays (fractionation by liquid-liquid partition, MIC determination, and phytochemical screening) because of its wide spectrum of antimicrobial activity. The MIC determination (Table 2) showed that leaf crude extracts also inhibited the microbial growth of all evaluated Gram-positive bacteria and yeasts (MIC ranging from 2.631 to 0.658 mg·mL^−1^), in agreement with the antimicrobial results by the previous ADD test. However, stem methanol extracts, although inactive in the ADD assay, showed action against *S. aureus* CCMB263 and *C. albicans*, in the latter with MIC values similar to those obtained by the leaf extracts. The reason for the difference in sensitivity between the two methods might be related to diffusion properties of the antimicrobial compounds from the extracts into the agar and adsorption in the paper disk [18].

The fractions from leaves and stems exhibited varying degrees of antimicrobial action, with hexane and ethyl acetate fractions from leaves showing the highest antibacterial effect against *S*. *aureus* (MIC—0.329 mg·mL^−1^). An enhanced activity against all microorganisms was observed with the fractionation, including against *E*. *coli*, to which the extracts were considered inactive. Dichloromethane fractions from leaves and stems, as well as the ethyl acetate fraction from leaves, were the most efficient against *E*. *coli *(MIC—0.658 mg·mL^−1^). The best result against *C*. *albicans *was obtained by the dichloromethane fraction from the stems (MIC—0.658 mg·mL^−1^), a noteworthy result considering that invasive candidiasis is a leading cause of mycosis-mortality [19].

The results of phytochemical screening of *L. origanoides* fractions showed the presence of terpenes, steroids, coumarins, saponins, flavonoids, and phenolic acids in stems and leaves, while alkaloids were absent (Table 3). These phytoconstituents, common in *Lippia* [5], are recognized as antimicrobial agents against several bacteria and yeasts [20]. Terpenes were the predominant metabolites in the hexane fractions and flavonoids in ethyl acetate fractions. These constituents may be the responsible for the improved antibacterial activity in hexane and ethyl acetate fractions. 

## 4. Conclusions

The antimicrobial activity by ADD gives preliminary scientific evidence to the traditional use of *L. alnifolia*, *L. origanoides,* and *L. thymoides* as antiseptic agents and in the treatment of infectious diseases. Although the results of this study suggest the antibacterial potential of three evaluated species, the *L. origanoides* leaves exhibited the widest antimicrobial action being a promising alternative in the treatment of bacterial and fungal infections. As the fractionation of *L. origanoides* crude extracts improved the activity in spectrum and intensity, further investigations are in progress to isolate and characterize the active metabolites.

## Figures and Tables

**Table 1:** Antimicrobial activity by disk diffusion method of crude extracts from *L. origanoides*, *L. alnifolia*,* L. thymoides*, and reference antibiotics.

Samples	Inhibition zones ± (mm)^1^					
	*E. coli* CCMB261	*S. aureus* CCMB262	*S. aureus* CCMB263	*P. aeruginosa* CCMB268	*C. albicans* CCMB286	*C. parapsilosis* CCMB288
*L. alnifolia* leaves	R	10.2 ± 1.3	10 ± 0.5	R	R	R
*L. alnifolia* stems	R	12.3 ± 1.0	13.9 ± 0.8	R	R	R
*L. alnifolia* flowers	R	8.4 ± 0.9	R	R	R	6.9 ± 0.1

*L. origanoides* leaves	R	13.0 ± 0.5	12.0 ± 0.9	R	11.7 ± 0.4	7.1 ± 0.4
*L. origanoides* stems	R	R	R	R	R	R

*L. thymoides * leaves	R	11.2 ± 1.0	9.7 ± 1.3	R	R	R
*L. thymoides *stems	R	8.7 ± 0.6	R	R	R	R
*L. thymoides * flowers	R	8.5 ± 1.5	R	R	R	R

Cloramphenicol^2^	15.8 ± 1.7	22.6 ± 2.2	R	8.5 ± 0.9	—	—

Nystatin^3^	—	—	—	—	17.5 ± 0.9	16.6 ± 0.9

^1^Inhibition zones including the diameter disk (6 mm); ^2^cloramphenicol at 30 *μ*g/disk; ^3^nystatin at 10 *μ*g/disk; R: resistant; (—): Not evaluated.

**Table 2:** Minimum inhibitory concentration (MIC) of the crude extract and fractions from *L. origanoides* and reference antibiotics.

Samples	Minimum inhibitory concentration (mg·mL^−1^)					
	*E. coli * CCMB261	*S. aureus * CCMB262	*S. aureus * CCMB263	*P. aeruginosa * CCMB268	*C. albicans* CCMB286	*C. parapsilosis* CCMB288
*L. origanoides* leaves						
CEXL	5.263	1.316	0.658	5.263	2.631	2.631
HEFL	1.316	—	0.329	—	2.631	—
DIFL	0.658	—	2.631	—	1.316	—
EAFL	0.658	—	0.329	—	2.631	—
AQFL	2.631	—	2.631	—	2.631	—

*L. origanoides* stems						
CEXS	5.263	5.263	2.631	5.263	2.631	5.263
HEFS	2.631	—	0.658	—	2.631	—
DIFS	0.658	—	2.631	—	0.658	—
EAFS	1.316	—	2.631	—	2.631	—
AQFS	5.263	—	5.263	—	2.631	—

Chloramphenicol	10	0.078	0.313	5	—	—

Nystatin	—	—	—	—	1.25	2.5

(—): not evaluated; CEX: crude extract; HEF: hexane fraction; DIF: dichloromethane fraction; EAF: ethyl acetate fraction; AQF: aqueous fraction; L: leaves; S: stems. MIC = 2.631 mg·mL^−1^ was the maximum value considered for attribution of antimicrobial activity for each strain.

**Table 3:** Phytochemical screening results of the fractions of *L. origanoides* stems and leaves.

Reagent	AS	DRG	LB	KOH	NP/PEG	
Metabolites	Terpenes and steroids	Alkaloids	Triterpenes and steroids	Coumarins and anthraquinones	Flavonoids and phenolic acids	Saponins
*L. origanoides* leaves						
HEFL	**++**	−	**+**	+	**+**	−
DIFL	**+**	−	**+**	+	**+**	−
EAFL	**+**	−	**+**	−	**++**	+
AQFL	**+**	−	**+**	−	**+**	+

*L. origanoides* stems						
HEFS	**++**	−	**+**	+	−	−
DIFS	**+**	−	**+**	−	**+**	−
EAFS	**+**	−	**+**	−	**++**	+
AQFS	+	−	+	−	**+**	+

AS: anisaldehyde sulfuric acid reagent; DRG: Dragendorff reagent; LB: Liebermann-Burchard reagent; KOH: potassium hydroxide reagent; NP/PEG: natural products/polyethylene glycol reagent; CEX: crude extract; HEF: hexane fraction; DIF: dichloromethane fraction; EAF: ethyl acetate fraction; AQF: aqueous fraction; L: leaves; S: stems; (−): not detectable; (+): present; (++): highly present.

## References

[1] WHO (2012). *The Evolving Threat of Antimicrobial Resistance—Options for Action*.

[2] Sibanda T, Okoh AI (2007). The challenges of overcoming antibiotic resistance: plant extracts as potential sources of antimicrobial and resistance modifying agents. *African Journal of Biotechnology*.

[3] Bussmann RW, Malca-García G, Glenn A (2010). Minimum inhibitory concentrations of medicinal plants used in Northern Peru as antibacterial remedies. *Journal of Ethnopharmacology*.

[4] Vogel NW, Taschetto APD, Dall’Agnol R, Weidlich L, Ethur EM (2011). Assessment of the antimicrobial effect of three plants used for therapy of community-acquired urinary tract infection in Rio Grande do Sul (Brazil). *Journal of Ethnopharmacology*.

[5] Pascual ME, Slowing K, Carretero E, Mata DS, Villar A (2001). Lippia: traditional uses, chemistry and pharmacology: a review. *Journal of Ethnopharmacology*.

[6] Oliveira DR, Leitão GG, Bizzo HR (2007). Chemical and antimicrobial analyses of essential oil of *Lippia origanoides* H.B.K. *Food Chemistry*.

[7] Funch LS, Harley R, Funch R (2004). *Plantas Úteis: Chapada Diamantina*.

[8] Agra MDF, Silva KN, Basílio IJLD, de Freitas PF, Barbosa-Filho JM (2008). Survey of medicinal plants used in the region Northeast of Brazil. *Brazilian Journal of Pharmacognosy*.

[9] dos Santos FJB, Lopes JAD, Cito AMGL, de Oliveira EH, de Lima SG, Reis FDAM (2004). Composition and biological activity of essential oils from *Lippia origanoides* H.B.K. *Journal of Essential Oil Research*.

[10] Ramírez LS, Isaza J, Veloza LA, Stashenko E, Marín D (2009). Actividad antibacteriana de aceites esenciales de *Lippia origanoides* de diferentes orígenes de Colombia. *Ciencia*.

[11] Tangarife-Castaño V, Correa-Royero J, Zapata-Londoño B, Durán C, Stanshenko E, Mesa-Arango AC (2011). Anti-*Candida albicans* activity, cytotoxicity and interaction with antifungal drugs of essential oils and extracts from aromatic and medicinal plants. *Infection*.

[12] Clinical and Laboratory Standards Institute (CLSI) (2003). *Performance Standards for Antimicrobial Disk Susceptibility Tests: Approved Standard, M2-A8*.

[13] Clinical and Laboratory Standards Institute (CLSI) (2003). *Methods for Dilution Antimicrobial Susceptibility Tests for Bacteria That Grow Aerobically: Approved Standard, M7-A6*.

[14] Clinical and Laboratory Standards Institute (CLSI) (2002). *Reference Method for Broth Dilution Antifungal Susceptibility Testing of Yeasts: Approved Standard, M27-A2*.

[15] Wagner H, Bladt S, Zgainsk EM (1984). *Plant Drug Analysis. A Thin Layer Chromatography Atlas*.

[16] Hoskins J, Alborn WE, Arnold J (2001). Genome of the bacterium *Streptococcus pneumoniae* strain R6. *Journal of Bacteriology*.

[17] Latgé J-P (2007). The cell wall: a carbohydrate armour for the fungal cell. *Molecular Microbiology*.

[18] Valgas C, de Souza SM, Smânia EFA, Smânia A (2007). Screening methods to determine antibacterial activity of natural products. *Brazilian Journal of Microbiology*.

[19] Pfaller MA, Diekema DJ (2007). Epidemiology of invasive candidiasis: a persistent public health problem. *Clinical Microbiology Reviews*.

[20] Cowan MM (1999). Plant products as antimicrobial agents. *Clinical Microbiology Reviews*.

